# Mental Research and Government Grants

**Published:** 1913-10-11

**Authors:** Joseph Shaw Bolton

**Affiliations:** Medical Director, West Riding Mental Hospital, Wakefield; Professor of Mental Diseases, University of Leeds


### MENTAL RESEARCH AND GOVERNMENT GRANTS.

### How Best to Assist Existing Laboratories,

By JOSEPH SHAW BOLTON, D.Sc., M.D., F.R.C.P., Medical Director, West Riding Mental Hospital, Wakefield; Professor of Mental Diseases, University of Leeds.

It might at first sight appear unnecessary to give any reasons in favour of Government grants in aid of mental research beyond the mere statement that the State controls the treatment of upwards of 138,000 notified insane persons under care in England and Wales, and ought naturally, therefore, to interest itself in the promotion of research with the object of curing, alleviating, or preventing insanity. The conditions of life in institutions devoted to the care of the insane are, however, so generally abnormal and unusual, and often react so undesirably on those responsible for the treatment of the mentally afflicted, as to provide additional and very important reasons why the Government should much more practically interest itself in the scientific training and practice of the medical staffs of mental hospitals.

### The Mental Hospital Service.

The special conditions of mental hospital life in this country have gradually evolved along the lines of least resistance during upwards of a century. During this period much that is good has been developed, particularly from the aspect of traditions and methods of humane treatment, and a number of brilliant investigators have, usually by their unaided efforts, carried out monumental work which has placed British psychiatry in a position of which it need not be ashamed, even in the face of the recent results of Continental and American research. It is nevertheless a lamentable truth that all is not as it should be in the case of the rank and file of the medical mental hospital service.

The English asylum medical officer usually receives no special training to fit him for his future career. He often, in fact, adopts psychiatry as a career after he has served long enough in a mental hospital to be already a failure at it or any other branch of medicine. The non-scientific routine work infallibly kills enthusiasm in all but the few, and the absence of responsibility in most mental hospitals, even to a pathologist, for reasonable correctness in diagnosis, together with the knowledge that the less expensive the treatment which is adopted the less is the disfavour which will result, tend to destroy interest in scientific medicine.

In too many mental hospitals, therefore, the average "chronic" assistant medical officer becomes eventually, at the one pole, a sort of county squire who enjoys life and takes his routine work easily and his responsibilities lightly, and, at the other, a confirmed, discontented, and dyspeptic hypochondriac who blames his committee, his superintendent, his conditions of service, his salary and opportunities—everything, in fact, but the lack of original training in scientific medicine and the absence of the necessary backbone to work and force his name to the front—for his hopeless position and prospects. The scientific training in psychological medicine of all assistant medical officers of mental hospitals who propose to make psychiatry their profession is in truth the only possible means by which alienism in this country may be removed from the slough of despond into which it has gradually and imperceptibly fallen.

Such training can obviously only be given at certain places—namely, in such of the mental hospitals as possess adequate equipment for teaching and research and suitably qualified teachers and workers. With regard to the practicability of this, it may be said that past experience has already rendered it abundantly clear that the efforts which have hitherto been made to found small schools of scientific psychiatry have been spasmodic and badly sustained, for the reason that they have resulted solely owing to the enthusiasm of certain celebrated alienists backed by the public-spirited action of their committees. As examples of districts which have done yeoman service for the advancement of English psychiatry—without wishing to disparage in any way the work done elsewhere—I will mention the West Riding of Yorkshire during the past half century, Lancashire and London for considerable but less periods of time, and the Cardiff Mental Hospital since its opening five years ago.

The great majority of alienists—and mental hospital committees—have, however, been supine. It is thus clear that what private enterprise has performed locally and more or less imperfectly—but nevertheless at times with conspicuous success—is never likely to become sufficiently extensive in range to leaven the whole of the profession of psychiatry unless some form of State aid, with as a corollary some degree of State advice and control, is provided. A State service for the treatment of the insane is not at present in contemplation and is not likely to be instituted during the present generation.

### The "Via Media" of Grants in Aid.

The training of assistant medical officers and the granting of diplomas in psychological medicine can, however, be carried out by the different Universities, *if it be made worth the while of the assistant medical officers of mental hospitals to submit to the necessary training.* This would rapidly become general if, after a certain date fixed by Parliament, the power of the committees of mental hospitals to appoint whomsoever they wished to the post of superintendent were qualified by the proviso that all candidates for the higher administrative posts must be fully qualified medical men possessing diplomas in psychological medicine. Such is, I fear, too much to hope for at present.

There remains, therefore, the imperfect method of the giving of Government grants in aid of research in mental disease. By this I mean more than merely the doling out of small sums here and there for the purchase of apparatus—more even than the partial financing of the laboratories of mental hospitals. What I plead for is a definite Government plan for the fostering of research, and the hall-marking of research-workers, together with its necessary adjunct the subsidising of competent teachers of psychiatry, and of suitably recommended assistant medical officers who desire study-leave for scientific training either to gain a diploma in psychological medicine at one of the Universities or to carry out researches in psychiatry.

### The Object of Research.

It is unnecessary here to produce arguments in favour of research in psychiatry, as on this necessarily depends all advance in the knowledge, treatment, and prevention of mental disease. I would, however, remark that startling discoveries, with revolutionary results, are as unlikely here as in any other field of research, though valuable and helpful conclusions are certain to be gained. The object of research is, however, not solely the discovery of new truths. The discovery of truths, great or small, is very important, but the verification and diffusion of these is equally, or even more, valuable. Till performed work is known and proved, and believed to be true, it is relatively or entirely valueless except in so far as all sound work locally fosters the scientific spirit. I may go further and say that no honest efforts at research are ever quite wasted, even if these remain unknown, in consequence of the important, because active, influence for good of workers on non-workers. A man whose individuality is such as to render him capable of working alone, in spite of the deadly apathy induced by mental hospital routine, is truly a man to encourage, and one worthy of State aid. How much more likely is such to do good work if he receives the opportunity of working in a suitable environment, and of receiving skilled assistance according to his needs, neither of which is at the present time usually possible without the sacrifice of his means of livelihood. Apart, however, from the actual amount of research work performed, the atmosphere of the scientific laboratory and the diffusion of knowledge by actual contact between workers and learners necessarily raises the standard and ideals of the latter, and makes them better men and women.

### The Application of State Grants.

Having as I hope made out a case for the granting of State aid for mental research, I will now proceed to indicate the purposes to which, in my opinion, such aid may be most usefully applied. These may be stated briefly as follows:—

(1) The subsidising of the special researches of proved workers;

(2) The part-payment of the salaries of the special pathologists of recognised laboratories;

(3) The subsidising of the special teaching of psychiatry in recognised laboratories to approved workers from other mental hospitals; and

(4) The subsidising of approved assistant medical officers who desire study-leave in order to work in a recognised mental hospital and laboratory.

The administration of such a scheme would be best carried out by the Board of Commissioners in Lunacy, since this body is responsible for the supervision of the treatment of the insane in England and Wales. The Commissioners in Lunacy, in fact, owing to their intimate knowledge not only of the conditions of mental hospital life, but also of the medical staffs of mental hospitals together with their scientific positions and capabilities, are obviously the government body who could best be trusted judicially and impartially to administer a Government grant for the furthering of such objects as I have indicated.

Certain mental hospitals already possess the reputation, equipment, and staff which fit them to become scientific centres of teaching and research under the auspices of the Commissioners in Lunacy; and the local authorities controlling others in suitable districts without doubt would readily make the necessary provision and accept the responsibilities and subsidies. In the first instance, no doubt, only such institutions as possessed a suitable staff would be recognised as scientific centres, for the Board of Commissioners in Lunacy could be trusted only to subsidise workers of merit and pathologists and teachers of recognised ability. Later, as increased facilities were needed, additional centres would be subsidised, subject not only to their pathologists, but also to their responsible administrators being men of proved scientific merit.

### The Guildhall Conference.

Such a plan would, at the outset, probably require a subsidy of a few thousands at the most, and as it evolved the subsidy might eventually rise perhaps to about £20,000 per annum, a small sum to pay for a really efficient medical service for the scientific study and treatment of nearly 140,000 insane persons, with the further object of indicating the best means by which the fell scourge of mental disease may be combated. As evidence that the above plan is within the range of practical politics, I would remark that a large and representative conference of the visiting committees of the mental hospitals of England and Wales was held at the Guildhall in December last, with the object of urging the need for State Aid in the shape of grants towards research for the prevention, cure, and treatment of mental disease. A deputation later on waited on the Commissioners in Lunacy; and in May last the Home Secretary paid a visit to the Cardiff Mental Hospital. The Commissioners in Lunacy, in their recently issued sixty-seventh report (pp. 62-3), refer sympathetically and at some length to this question, and indicate, as purposes to which State Aid might legitimately be given, the two to which I have accorded the first place on my list. The Commissioners then remark: "An ideal to be aimed at in this connection is that of the establishment of central laboratories or institutes (possibly associated with psychiatric clinics) with each of which several asylums might be affiliated; and, since this would involve the co-operation of local authorities, the question of legislative sanction must necessarily arise, as well as that of determining the share which might well be taken by the State in the promotion of a scheme of truly national importance.

The Limits of Affiliation.

With all respect, I would suggest that even legislative sanction would not in my opinion render successful an attempted affiliation of different county and county borough authorities for the purpose of providing central laboratories or institutes. Would the Claybury Laboratory, for example, have been founded had not all the affiliated asylums been under one asylums committee? And would the laboratory have been such a conspicuous success, or even been now existent, had it had to depend on the co-operation of the different mental hospital staffs rather than on the administrative genius of its celebrated director?

The main purpose of this article is to indicate how existing laboratories could be assisted, and by their multiplication eventually made a success from the aspects both of special research and of the scientific training of the staffs of mental hospitals, without any attempt at a co-operation of separate local authorities under legislative sanction. In other words, I suggest that the road to success lies in the co-operation of the State and such different local authorities as have voluntarily provided, or would provide, suitable laboratories and staff; and not in the attempted affiliation of local authorities into groups with central laboratories and staffs, sanctioned and partly supported by the State. In order to avoid being misunderstood I ought to remark here that the asylums boards of Lancashire and of the West Riding of Yorkshire should be regarded for my purpose as single local authorities.

Units for Research.

I will now briefly elaborate my views regarding the activities of such different scientific centres as exist, or would be established or subsidised throughout the country, under such a scheme as I have outlined. Each of these should be regarded, in my opinion, as a kind of "hospital unit" for research and training in psychiatry. At the beginning, and probably later, no two centres would have the same aims and ideals, or even a similar equipment for their work. Some centres would lay themselves out chiefly or entirely for advanced research in special departments, *e.g.*, biochemistry, experimental psychology, etc. Others would largely combine teaching with research in morbid histology, bio-chemistry, bacteriology, etc. Others again would go in chiefly for the training of the assistant medical officers of more or less neighbouring mental hospitals for university diplomas in psychiatry, and would do relatively little pure research, but probably carry on and record a large amount of useful routine pathological work.

I suggest no attempt to co-ordinate or generally to control the work of these several units. Individuality and originality in research are essential if this is to be of value. Spade-work, it is true, can be done by anyone; but what master of research needs an army of labourers, or could obtain them, unless they were women, since the female sex is, as a whole, more industrious and less original than the male? Research workers of individuality and originality, though at first they would be willing and anxious to work under the guidance of a leader, would sooner or later throw off their shackles, even though these were forged by the greatest genius the world could produce. In time, no doubt, as our knowledge increases, this will gradually become more and more co-ordinated, as has already occurred in the past; but great advances cannot be made by research work done in bulk or to order.

The Case against Co-ordination.

The brains behind the work, with the individuality and originality which spell success, are the real source of the results obtained by research. To reason that such work may be done by many, but at the same time co-ordinated and controlled by a master mind, would be to misunderstand the elements of success. The scientific worker is at once an aristocrat and a socialist—but is never a syndicalist—since the one article of his creed is that all men are equal except in brains, and that, therefore, any master may become a servant, and any servant a master. Further, the greatest of scientific centres would rapidly fall from its high estate if the men of brains forsook it, since they, and not their laboratory equipment or financiers, are the source of the knowledge and benefits which result from research.

It is, therefore, essential, in my opinion, that no general co-ordination of research work should be attempted, and that the different voluntary centres of research in the scheme I have described should be left to vie with one another for the advancement of psychiatry, as have done the fewer number of working areas in the past.

